# Tinostamustine–nivolumab therapy is associated with antigen-specific cellular and humoral immune responses in heavily pre-treated patients with advanced melanoma

**DOI:** 10.1007/s00262-026-04608-4

**Published:** 2026-11-05

**Authors:** Shrunal Mane, Vincent Walter, Marie-Therese Abdou, Stefan Diem, Nina Wyss, Kira-Lee Koster, Lenka Besse, Dagmar Hess, Yannis Metaxas, Marie-Claire Flynn, Stefanie Aeppli, Taemer El Saadany, Elke Hiendlmeyer, Roger von Moos, Markus Joerger, Lukas Flatz

**Affiliations:** 1 Department of Dermatology, University Hospital, Liebermeisterstraße 20, 72076 Tübingen, Germany; 2 Department of Dermatology, Institute of Immunobiology, St. Gallen, Switzerland; 3 Department of Medical Oncology and Hematology, Cantonal Hospital, St. Gallen, Switzerland; 4 Department of Internal Medicine, Regional Hospital, Grabs, Switzerland; 5 Department of Dermatology, Cantonal Hospital, St. Gallen, Switzerland; 6 Department of Oncology and Hematology, Laboratory of Experimental Oncology, Cantonal Hospital, St. Gallen, Switzerland; 7 Department of Biology, Faculty of Medicine, Masaryk University, Brno, Czech Republic; 8 Department of Oncology/Hematology, Spital Thurgau, Münsterlingen, Switzerland; 9 Cancer Center, Cantonal Hospital Graubünden, Chur, Switzerland; 10 Division of Oncology/Hematology, Cantonal Hospital Graubünden, Chur, Switzerland; 11 Clinical Trial Unit (CTU), Cantonal Hospital, St. Gallen, Switzerland; 12 Medical School, University Hospital, Basel, Switzerland

**Keywords:** Melanoma, Tinostamustine, Histone deacetylase (HDAC) inhibitors, Nivolumab, Heavily pre-treated patients

## Abstract

ENIgMA is a phase IB trial (NCT03903458; SNCTP000003243) evaluating the safety and preliminary activity of the first-in-class alkylating deacetylase inhibitor tinostamustine in combination with nivolumab (3 mg/kg) in patients with predominantly immune checkpoint inhibitor (ICI)-resistant, advanced melanoma. We conducted this longitudinal immune monitoring study in 15 of the 17 enrolled patients to characterize systemic immunologic changes associated with an immunomodulatory dose of tinostamustine. We have previously demonstrated that combination of tinostamustine with nivolumab was able to induce disease stabilization in 54% and confirmed partial responses in 23% of evaluable patients. Peripheral blood samples collected longitudinally during treatment were analysed by flow cytometry, serum antigen-specific IgG enzyme-linked immunosorbent assay (ELISA), and intracellular cytokine staining following ex vivo peptide stimulation. Longitudinal flow cytometric analyses demonstrated dynamic changes in peripheral immune subsets, including T cells, natural killer (NK) cells and monocytes, suggesting ongoing immune engagement during tinostamustine–nivolumab therapy. In progressors, elevated baseline lactate dehydrogenase (LDH) levels significantly and inversely correlated with circulating T cell frequencies, indicating distinct immune profiles associated with poor clinical outcome. Antigen-specific adaptive immune responses were detectable despite extensive prior treatment and high tumour burden. CD4+ T cell responses, particularly TNFα-associated responses directed against melanoma-associated antigens, represented the dominant cellular immune activation pattern. Additionally, treatment related increases in melanoma-associated antigen-specific IgG responses were also observed in several patients. Collectively, these findings demonstrate that immunomodulatory doses of tinostamustine in combination with nivolumab are linked with preserved adaptive immune competence and detectable cellular and humoral immune activation in heavily pre-treated patients with advanced melanoma.

**Supplementary Information:**

The online version contains supplementary material available at https://doi.org/10.1007/s00262-026-04608-4.

## Introduction

Advanced melanoma is a highly aggressive malignancy historically characterized by a poor prognosis [1]. Treatment with immune checkpoint inhibitors (ICIs) has significantly improved clinical outcome in patients with unresectable or metastatic disease [2, 3]. However, a considerable proportion of patients either fail to respond or eventually develop resistance to systemic treatment including ICIs. Accordingly, there is a substantial unmet need to develop novel therapeutic strategies that are capable of enhancing anti-tumour immunity while maintaining tolerability [4, 5].

Tinostamustine (EDO-S101) is a novel, first-in-class alkylating histone deacetylase inhibitor with improved access to cancer cell DNA, resulting in DNA damage and impairment of DNA repair mechanisms [6]. Tinostamustine’s anti-tumour activity has been demonstrated across multiple preclinical tumour models, where it induces genotoxic stress, increases chromatin accessibility, and renders tumour cells more susceptible to immune-mediated killing [7]. In an in vitro model of multiple myeloma, tinostamustine was shown to increase tumour immunogenicity by upregulating CD38 and the NK cell ligands, major histocompatibility complex class I chain-related proteins A and B (MICA and MICB), thereby enhancing sensitivity to CD38-targeting antibody-mediated cytotoxicity. Furthermore, tinostamustine pre-treatment synergized with daratumumab to delay tumour growth and prolong survival in a myeloma mouse model [8, 9]. These findings suggest that beyond its direct cytotoxic effects, tinostamustine may modulate the tumour microenvironment and synergize with ICIs. The ENIgMA trial was initiated to investigate immunomodulatory doses of tinostamustine in combination with nivolumab in patients with predominantly relapsed, incurable melanoma. In this phase IB trial, tinostamustine was administered intravenously every two weeks at a dose of 15 mg/m^2^ (for four of 17 enrolled patients) and 30 mg/m^2^ (for thirteen of 17 enrolled patients) over 60 mins for the first cycle, and in combination with nivolumab (3 mg/kg) from cycle two on [10]. The combination of tinostamustine and nivolumab was well tolerated and demonstrated encouraging clinical activity, with over half of the patients (54%) achieving disease stabilization and 23% exhibiting confirmed partial responses. Notably, the trial cohort consisted of 77% (13/17) of individuals with ICI-resistant advanced melanoma [10].

Although clinical activity and safety findings from the ENIgMA trial have previously been reported, the immunologic mechanisms underlying these observations remain poorly understood. Conventional clinical parameters and tumour burden provide limited insight into dynamic immune changes related to study treatment [11, 12]. On the other hand, longitudinal translational immune profiling offers the opportunity to assess whether heavily pre-treated melanoma patients retain the capacity to mount detectable adaptive immune responses to study treatment. In particular, characterization of antigen-specific T cell activation and melanoma-associated humoral immune responses may provide important insights into the involvement of T cells and B cells during therapy. Here, we describe the results of a longitudinal translational study to determine whether immunomodulatory doses of tinostamustine are capable of inducing detectable adaptive cellular and humoral immune responses in predominantly pre-treated advanced melanoma patients and to identify peripheral immune patterns associated with clinical outcome. The translational cohort described in this study includes fifteen of the seventeen patients that were enrolled in the ENIgMA trial.

## Materials and methods

### Patient cohort

ENIGMA (NCT03903458; SNCTP000003243) is an open-label, multicentre phase IB study conducted at two sites in Switzerland. The study protocol was approved by the involved competent ethics committees and Swissmedic. The clinical trial was conducted in accordance with the approved protocol, the Declaration of Helsinki, ICH-GCP guidelines, and all applicable national legal and regulatory requirements. All participants provided written informed consent prior to study enrolment. We included patients with histologically or cytologically confirmed, unresectable locally advanced or metastatic melanoma ineligible for first-line combined programmed death-(ligand) 1 PD-(L)1/cytotoxic T-lymphocyte-associated protein 4 (CTLA4)-targeted treatment or progressing on prior standard palliative systemic treatments including ICI and/or BRAF/MEK-targeted tyrosine kinase inhibitors and measurable or evaluable disease according to RECIST version 1.1. Treatment consisted of tinostamustine monotherapy in the first cycle followed by combined tinostamustine and nivolumab from cycle 2 on. Study treatment was continued until disease progression, unacceptable toxicity or withdrawal of consent. Patients were followed for a minimum of 100 days after treatment discontinuation or until disease progression. The present translational analysis includes 15 of the 17 enrolled patients.

### Sample collection

Blood was collected prior to study treatment at predefined sampling timepoints: baseline (C1), 2 weeks following tinostamustine monotherapy (C2), 2 weeks after the first combination treatment (C3) and continued at 2-week intervals through cycle 10 (C10). At each timepoint, a total of 60 mL of peripheral venous blood was collected into Na-heparin tubes for peripheral blood mononuclear cells (PBMC) isolation, along with 2 serum tubes. All samples were processed within 8 h of collection to preserve cell viability and protein integrity. Standardized processing protocols were applied to minimize inter-sample variability.

### PBMC isolation and serum preparation

For isolation of PBMCs, collection tubes were centrifuged at 1650 × g for 20 min at room temperature. Following centrifugation, the supernatant containing PBMC layer was harvested, washed, and cryopreserved in fetal bovine serum with 10% DMSO at − 150 ℃ until analysis. For serum collection, up to 2 clot-activator tubes were drawn at each timepoint. Tubes were allowed to clot at room temperature for 30–60 min and then centrifuged at 500 × g for 15 min. Serum was aliquoted and stored at − 80 ℃ until analysis.

### Immune profiling

Single-cell suspensions of PBMCs were incubated in PBS containing 2% BSA and 0.01% NaN_3_ with the immune profiling antibody panel for 20 min at 4 ℃. The antibody panel included APC/Fire^TM^ 750 anti-human CD45 (clone HI30), Brilliant Violet 570^TM^ anti-human CD3 (clone UCHT1), Brilliant Violet 605^TM^ anti-human CD16 (clone 3G8), Brilliant Violet 750^TM^ anti-human CD56 (clone HCD56), Pacific Blue^TM^ anti-human CD20 (clone 2H7), PE/Fire^TM^ 810 anti-human HLA-DR (clone L243), Spark Blue^TM^ 550 anti-human CD14 (clone M5E2), and Spark NIR™ 685 anti-human CD19 (clone HIB19) (all BioLegend) used at a 1:100 dilution. For discrimination between live and dead cells, single-cell suspensions were stained with Zombie NIR^TM^ dye in PBS prior to antibody staining. Additionally, Human TruStain FcX was used to avoid false positives or false negatives as a result of FcRs-mediated Ig Fc binding. Cells were acquired with a Cytek® Aurora and data analysis was performed using FlowJo (version 10.6.2).

### ELISA for melanoma-associated antibodies

Serum IgG antibodies against melanoma-associated antigens: glycoprotein 100 (gp100), cancer/testis antigen 1B (NY-ESO-1), tyrosinase-related protein 1 and 2 (TRP1, TRP2) and melanoma antigen recognized by T cells 1 (MART1/MelanA) were quantified by ELISA. For gp100, TRP2 and NY-ESO-1, MediSorp flat-bottom 96-well plates (Thermo Fisher Scientific, 467320) were coated overnight at 4 ℃ with 100 ng of recombinant protein diluted in 50 mM carbonate buffer (pH 9.5). Plates were washed once with phosphate-buffered saline (PBS) containing 0.02% Tween-20 and blocked for 2 h at room temperature (RT) using MonsterBlock (ImmunoChemistry Technologies, ICT-6295). Freshly thawed serum samples were diluted 1:100 in PBS supplemented with 10% normal goat serum (Cell Signaling Technology, 5425S), added in duplicate (100 µL per well), and incubated for 1 h at RT with gentle agitation. Plates were subsequently washed six times with PBS containing 0.02% Tween-20.

For TRP1, plates were coated overnight at 4 ℃ with 100 ng recombinant protein in 50 mM carbonate buffer (pH 9.5), washed once with deionized water containing 0.02% Tween-20, and blocked for 4–6 h at RT using 1× ELISA Diluent (eBioscience, 00–4202-56). Serum samples were diluted 1:50 in PBS containing 5% non-fat dry milk, added in duplicate, and incubated overnight at 4 ℃ with agitation. Plates were then washed six times with PBS containing 0.02% Tween-20.

For MelanA, plates were coated with 100 ng recombinant protein in PBS (pH 7.4) for 2 h at 37 ℃, followed by washing and blocking for 2 h at RT with PBS-M. Serum samples diluted 1:50 in PBS-M were added in duplicate and incubated for 1 h at RT with agitation. Plates were washed six times with PBS containing 0.02% Tween-20.

For all assays, bound IgG was detected using horseradish peroxidase (HRP) conjugated goat anti-human IgG (H+L) (Jackson ImmunoResearch, 109–035-00) diluted 1:2000 in PBS containing 10% normal goat serum and incubated for 1 h at RT. After 6 washing steps, plates were developed using TMB substrate (Mabtech, 3652-F10). The reaction was stopped after 5 min by addition of 2.5 N sulfuric acid (Sigma-Aldrich, 320501-1), and absorbance was measured at 450 nm using a Sunrise microplate reader (Tecan).

### T cell stimulation with peptide pools

PBMCs were cultured in low-adherence 24-well plates (Sarstedt, 83.3922.500) at approximately 1 × 10^6^ cells per well in RPMI-1640 medium containing L-glutamine, supplemented with 8% human serum (Biowest, S4190-100), 1% penicillin/streptomycin, 1% non-essential amino acids, 1% sodium pyruvate, 0.1 mg/mL kanamycin, and 0.1% 2-mercaptoethanol. On the following day, PBMCs were stimulated with melanoma-associated antigen-specific peptide pools including MelanA, tyrosinase (TYR), gp100, TRP2, melanoma-associated antigens (MAGE) -A1, MAGE-A3, and MAGE-A4 (PepMix™ Melanoma Collection, JPT, PM-C-Mel), as well as NY-ESO-1 (PepMix™ Human, JPT, PM-NYE), at a final concentration of 2 µg/mL per peptide. Cells were expanded for 10 days, with medium supplemented with recombinant human IL-2 (150 U/mL). Following expansion, cells were restimulated with the corresponding peptide pools (2 µg/mL per peptide) in the presence of brefeldin A (10 µg/mL) for 6 h at 37 ℃ and 5% CO_2_. In parallel, cells were restimulated with a CEF peptide pool (derived from cytomegalovirus, Epstein–Barr virus and influenza virus) as a positive control and cultured without peptides (medium only) as a negative control. Cells were subsequently stained for viability using Zombie Aqua™ Fixable Viability Dye (BioLegend), followed by surface staining with antibodies against APC/Cy7 anti-human CD3 (clone OKT3), PE/Cy7 anti-human CD4 (clone OKT4), PerCP anti-human CD8 (clone SK1) (each 1:100), and Brilliant Violet 711™ anti-human CD45RA (clone HI100) (0.25:100), (all BioLegend), diluted in FACS buffer (PBS containing 10 mM EDTA, 2% foetal calf serum, and 0.05% sodium azide). Cells were then fixed and permeabilized using BD Cytofix/Cytoperm (BD, 51-2090KZ), followed by intracellular staining for PE anti-human IFN-γ (clone B27, eBioscience) and APC anti-human TNF-α Antibody (clone Mab11, BioLegend) (1:100) in permeabilization buffer (PBS supplemented with 2% foetal calf serum, 5 mM EDTA, 0.1% saponin, and 0.1% sodium azide). Data were acquired on a BD LSR Fortessa flow cytometer on the same day and analysed using FlowJo software (version 10.6.2). Lymphocytes were gated by forward and side scatter, followed by exclusion of doublets and dead cells. Non-naïve T cells were defined as CD3+ CD45RA-low/negative and divided into CD4+ and CD8+ subsets, within which IFNγ+ and TNFα+ cells were identified. Frequencies are reported as the percentage of cytokine-positive cells within the respective CD4+ or CD8+ non-naïve T cell population. All reported frequencies were background-corrected by subtracting the corresponding unstimulated medium-control response from each peptide-stimulated condition. The representative gating strategy, including the positive and negative controls, is provided as Supplementary Fig. S1.

### Data visualization and statistical analysis

The primary objective of this translational study was to determine whether heavily pre-treated melanoma patients retained the capacity to mount detectable adaptive immune responses when receiving tinostamustine–nivolumab treatment. Radiological response was assessed using RECIST v1.1. Patients achieving a partial response (PR, *n* = 3) or stable disease (SD, *n* = 4) were classified as non-progressors (NP), whereas patients with progressive disease (PD, *n* = 8) were classified as progressors (P). Peripheral blood parameters including routine haematology and inflammatory markers, immune cell subsets, serum antigen-specific IgG levels and functional antigen-specific T cell responses, were assessed over trial treatment and up to cycle 10. Mixed-effects models using restricted maximum likelihood estimation (REML) were applied to evaluate fixed effects of treatment cycle, response group, and cycle-by-response interaction while accounting for repeated measurements and missing values. Correlations between baseline tumour burden, LDH, S100 and peripheral immune cell frequencies were separately assessed in non-progressors and progressors using Spearman rank correlation analysis. For antigen-specific T cell stimulation assays, intracellular cytokine-producing CD4+ and CD8+ T cell frequencies following ex vivo peptide stimulation were quantified over treatment cycles. Treatment-associated changes were calculated relative to baseline as ΔC2–C1 and ΔC3–C1 and visualized as patient-level response heatmaps using a diverging colour scale that shows both increases and decreases relative to baseline. Similarly, longitudinal changes in serum melanoma antigen-specific IgG responses were calculated relative to baseline and visualized as heatmaps of Δ IgG OD450 values. A swimmer plot was used to display treatment duration, best response, the number of prior treatment lines and the timing of prior anti-PD-1 therapy for each patient; anti-PD-1 exposure was classified as none (anti-PD-1 naïve), immediate (anti-PD-1 was the line directly preceding study treatment) or remote (anti-PD-1 was received earlier, with at least one intervening line). To explore the influence of prior treatment, immune cell subsets, antigen-specific IgG and antigen-specific T cell responses were compared descriptively between treatment-naïve (*n* = 4) and previously treated (*n* = 11) patients and well as individual resist classification PR, SD and PD. Owing to the small size of the additional subgroups, no formal statistical testing was performed. Dates of disease progression and the prior anti-PD-1 treatment history of each patient are provided in Supplementary Table S1. All data processing, statistical analyses, and figure generation were performed in R version 4.5.0. Statistical significance was defined as *p* < 0.05.

## Results

### Patient characteristics

A total of 17 patients with advanced melanoma were enrolled at two clinical sites in Switzerland between May 2019 and April 2022. By the time of database lock (April 2024), all patients had completed study treatment. Two patients discontinued therapy within the first four weeks due to early disease progression. Of the 17 patients enrolled, PBMCs and serum samples suitable for immune monitoring were available for 15 participants, representing the translational analysis cohort described in this study. Baseline demographic and clinical characteristics for the 15 patients are summarized in Table 1. 4/15 patients received tinostamustine at 15 mg/m^2^ over 60 min, while the remaining 11/15 were treated at 30 mg/m^2^ over 60 min. The mean duration of treatment was 23.5 weeks. The two patients who were not included were both treated at 30 mg/m^2^. Best response by RECIST was partial response (PR) in 3/15 patients, stable disease (SD) in 4/15 and progressive disease (PD) in 8/15. 4/15 patients (26.7%) had received no prior palliative systemic therapy (treatment-naïve), and 11/15 (73.3%) had received at least one prior line. 10/15 patients (66.7%) had received prior anti-PD-1 therapy, which was the immediate prior line in 5/15 (33.3%) and a more remote exposure, with at least one intervening line, in 5/15 (33.3%).

**Table 1:** Baseline demographics and clinical characteristics of patients of the translational analysis cohort (N = 15)

Characteristic	Total (N = 15)
*Age (years)*	
Median (range)	68 (47–85)
> 65 years	9 (60%)
*Gender*	
Male	7 (46.7%)
Female	8 (53.3%)
*Melanoma subtype*	
Non-acral cutaneous	9 (60%)
Acral lentiginous	2 (13.3%)
Conjunctival	1 (6.7%)
Uveal	3 (20%)
*Mutation status*	
BRAF-mutant	3 (20%)
NRAS-mutant	6 (40%)
GNAQ/GNA11-mutant	2 (13.3%)
Triple wild-type (BRAF/NRAS/cKIT wt)	4 (26.7%)
*Prior lines of palliative treatment*	
0	4 (26.7%)
1	4 (26.7%)
2	3 (20%)
≥ 3	4 (26.7%)
*Prior treatment received*	
Treatment-naïve	4 (26.7%)
Prior anti-PD-1 therapy	10 (66.7%)
Immediate prior line	5 (33.3%)
Remote exposure	5 (33.3%)
Prior CTLA-4-based therapy	8 (53.3%)
Any prior ICI	10 (66.7%)
Prior BRAF/MEK-targeted therapy	1 (6.7%)
Prior chemotherapy	5 (33.3%)
Other prior immunotherapy (TIL, T-VEC)	1 (6.7%)
*Tinostamustine dose level*	
15 mg/m^2^	4 (26.7%)
30 mg/m^2^	11 (73.3%)
*Median number of cycles*	
Tinostamustine	8
Nivolumab	7
Mean treatment duration (weeks)	23.5
*Response by RECIST*	
Partial response (PR)	3 (20%)
Stable disease (SD)	4 (26.7%)
Progressive disease (PD)	8 (53.3%)

### Longitudinal blood immune marker dynamics shows no uniform cohort wide changes during *tinostamustine–nivolumab*

**Fig. 1:**
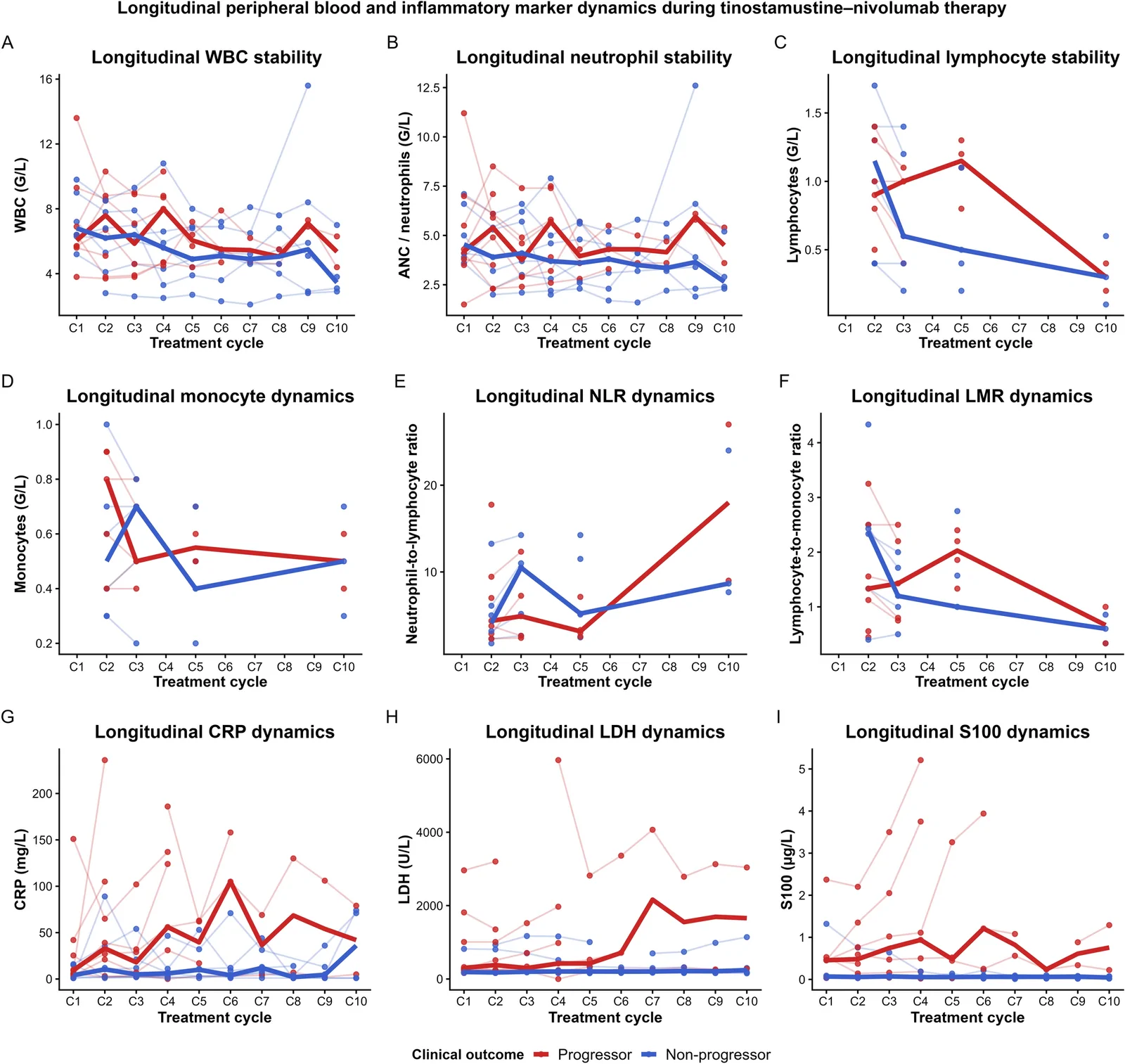
Longitudinal blood immune and inflammatory marker dynamics during tinostamustine–nivolumab therapy

Overall leukocyte and neutrophil counts did not change during treatment in both non-progressors and progressors (Fig. 1A, B). No consistent depletion or expansion trends were apparent across the cohort for absolute lymphocyte, absolute monocyte counts as well as Neutrophil-to-lymphocyte ratio (NLR) and Lymphocyte-to-monocyte ratio (LMR) analysis, suggesting an overall preservation of peripheral immune cell composition during therapy (Fig. 1C–F). In contrast, inflammatory and tumour burden-associated biomarkers demonstrated clearer response associated patterns. Progressors generally exhibited persistently elevated C-reactive protein (CRP), lactate dehydrogenase (LDH), and S100 levels throughout treatment compared with non-progressors (Fig. 1G–I).

Peripheral blood counts and inflammatory/tumour burden-associated markers were longitudinally assessed across treatment cycles in non-progressors (blue) and progressors (red) receiving tinostamustine–nivolumab therapy. Thin lines represent individual patients, whereas thick lines indicate group-wise mean trends over time. (A) White blood cell (WBC) counts. (B) Absolute neutrophil counts (ANC). (C) Absolute lymphocyte counts. (D) Absolute monocyte counts. (E) Neutrophil-to-lymphocyte ratio (NLR). (F) Lymphocyte-to-monocyte ratio (LMR). (G) C-reactive protein (CRP). (H) Lactate dehydrogenase (LDH). (I) S100 levels*.*

### Peripheral immune cell dynamics reveal active role of T cells and monocytes in disease progression

**Fig. 2:**
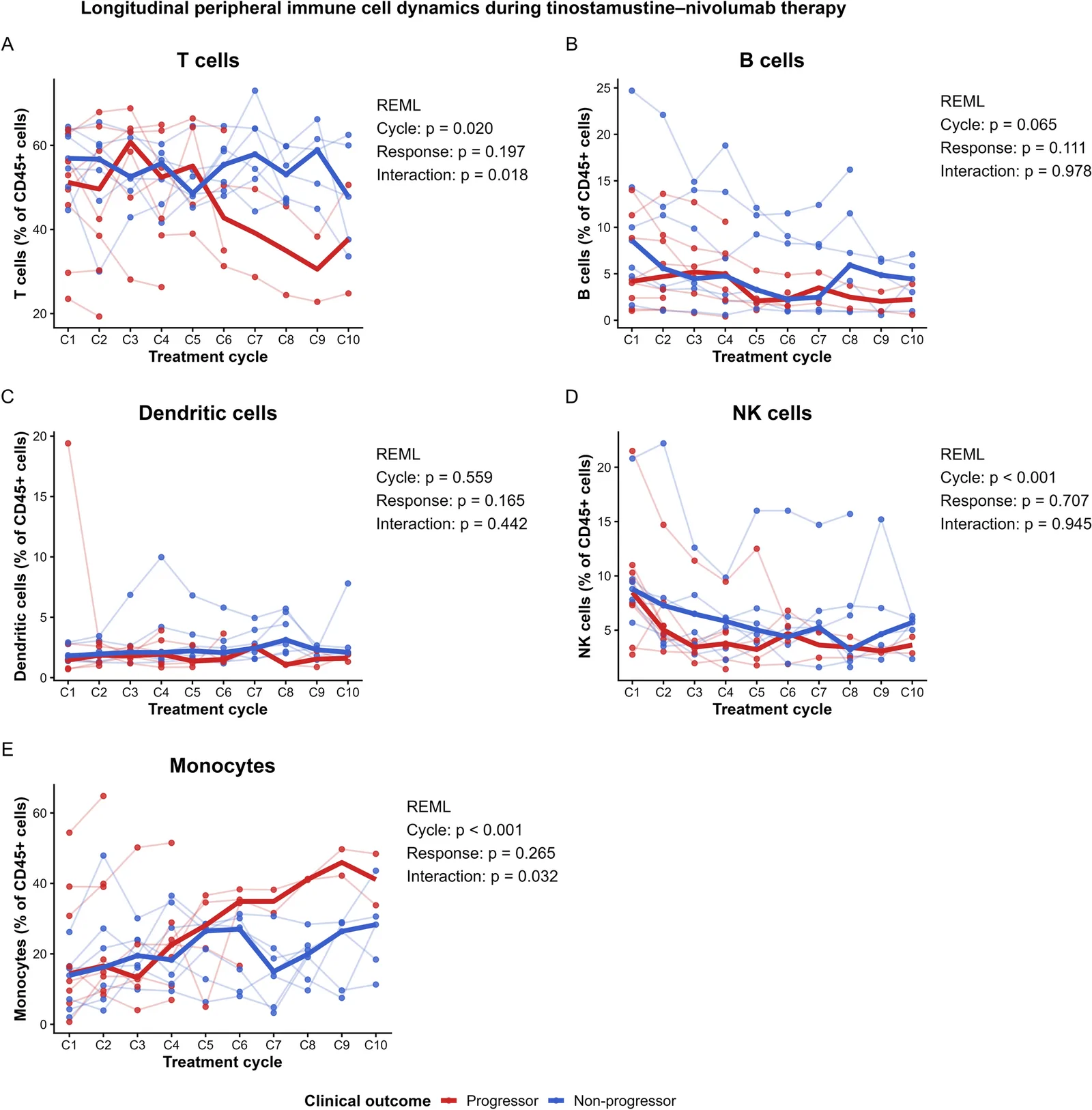
Longitudinal dynamics of peripheral immune cell populations during tinostamustine–nivolumab therapy

Following flow cytometric analyses of major peripheral immune cell populations performed across treatment cycles in non-progressors versus progressors (Fig. 2), longitudinal mixed-effects modelling (REML) demonstrated significant cycle-associated changes in selected immune subsets. T cell frequencies showed a significant temporal effect across treatment cycles (REML cycle effect, *p* = 0.020), while NK cells (*p* < 0.001) and monocytes (*p* < 0.001) similarly demonstrated dynamic longitudinal changes during therapy (Fig. 2A, D, E). T cells and monocytes additionally demonstrated significant cycle and response interactions, indicating distinct longitudinal immune trajectories between non-progressors and progressors. T cell frequencies progressively declined after C5 (10 weeks) in progressors (interaction *p* = 0.018; Fig. 2A). Conversely, monocyte frequencies increased over the course of therapy predominantly in progressors (interaction *p* = 0.032; Fig. 2E). No significant changes were observed for B cells and dendritic cells. (Fig. 2B, C). Trajectories for PR (*n* = 3), SD (*n* = 4) and PD (*n* = 8) are shown separately in Supplementary Fig. S3. Treatment-naïve (*n* = 4) and pre-treated (*n* = 11) patients showed broadly similar trajectories for all subsets, although NK cell frequencies tended to be higher at baseline in treatment-naïve patients (Supplementary Fig. S6).

Longitudinal flow cytometric analyses of major peripheral immune cell populations were performed across treatment cycles in non-progressors (blue, *n* = 7) and progressors (red, *n* = 8) receiving *tinostamustine–nivolumab* therapy. Thin lines represent individual patients, whereas thick lines indicate group-wise mean trends over time. Statistical analyses were performed using restricted maximum likelihood (REML) mixed-effects modelling. (A) T cells. (B) B cells. (C) Dendritic cells. (D) Natural killer (NK) cells. (E) Monocytes.

### Elevated baseline LDH levels are correlated to reduced peripheral T cell frequencies and increased monocyte abundance in progressors

**Fig. 3:**
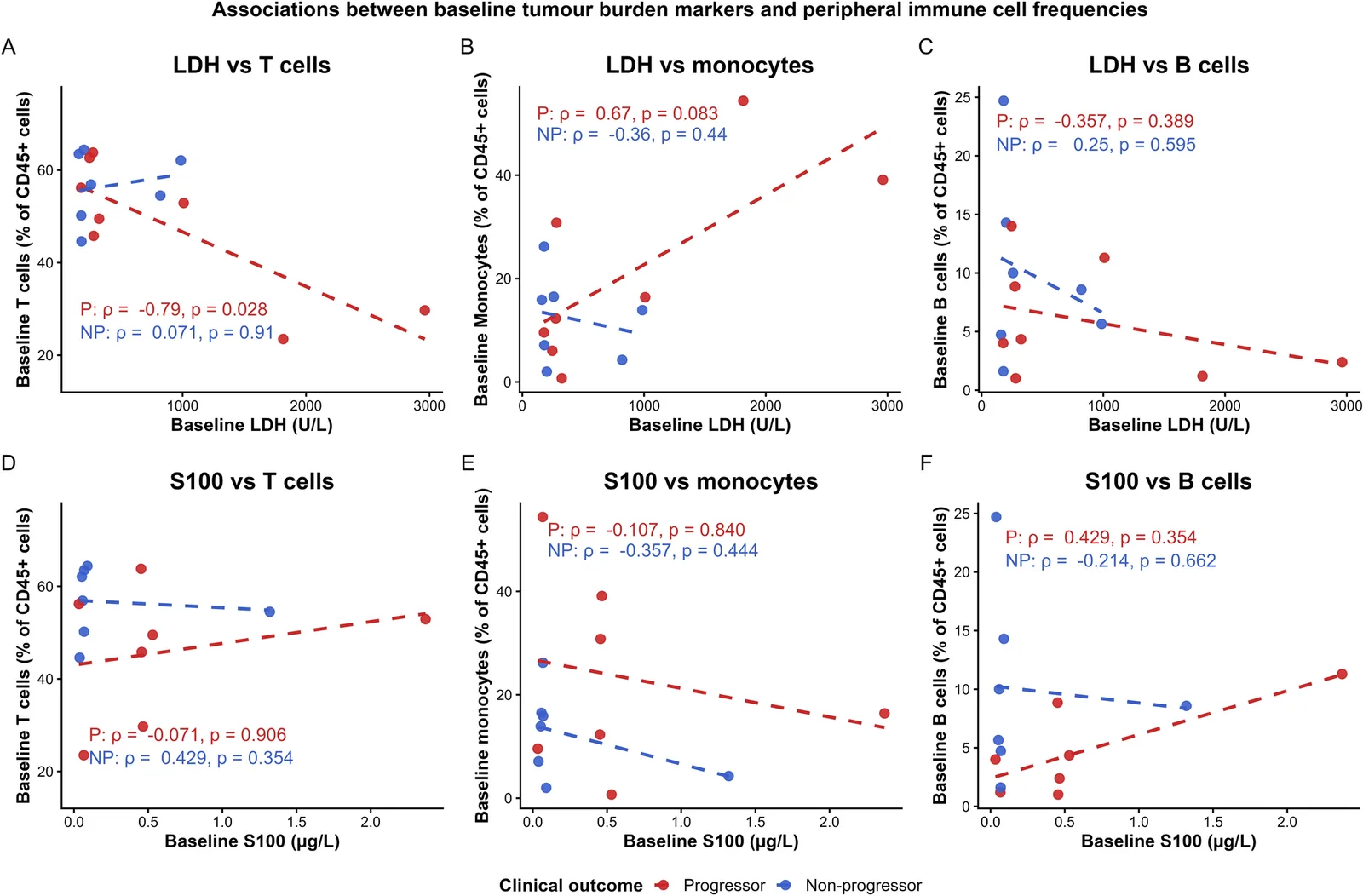
Correlation between baseline tumour burden markers and peripheral immune cell populations

Correlations between peripheral immune cell frequencies and melanoma-associated tumour burden markers (LDH and S100) were assessed separately in non-progressors (NP) and progressors (P) (Fig. 3). In progressors, elevated baseline LDH levels demonstrated a significant inverse correlation with circulating T cell frequencies (Spearman *ρ* = –0.79, *p* = 0.028), whereas no such association was observed in non-progressors (*ρ* = 0.071, *p* = 0.91) (Fig. 3A). These findings suggest that increased tumour burden in progressive disease is associated with reduced peripheral T cell abundance. Baseline LDH levels showed a positive correlation trend with circulating monocyte frequencies in progressors (Spearman *ρ* = 0.67, *p* = 0.083), although this did not reach statistical significance (Fig. 3B). No consistent associations between LDH and B cell frequencies were observed in either response group (Fig. 3C). Baseline S100 levels did not demonstrate significant correlations with peripheral T cell, monocyte, or B cell frequencies in either non-progressors or progressors (Fig. 3D–F).

Correlation analyses between baseline tumour burden-associated biomarkers and peripheral immune cell frequencies were performed separately in non-progressors (NP, blue) and progressors (P, red). Spearman correlation coefficients rho (ρ) and corresponding p values are shown within each panel. Dashed lines represent linear trend lines. (A-C): Correlation between baseline LDH levels and peripheral T cell, monocyte and B cell frequencies, respectively. (D-F): Correlation between baseline S100 levels and peripheral T cell, monocyte and B cell frequencies, respectively.

### Tinostamustine–nivolumab treatment is associated with antigen-specific peripheral T cell responses

**Fig. 4:**
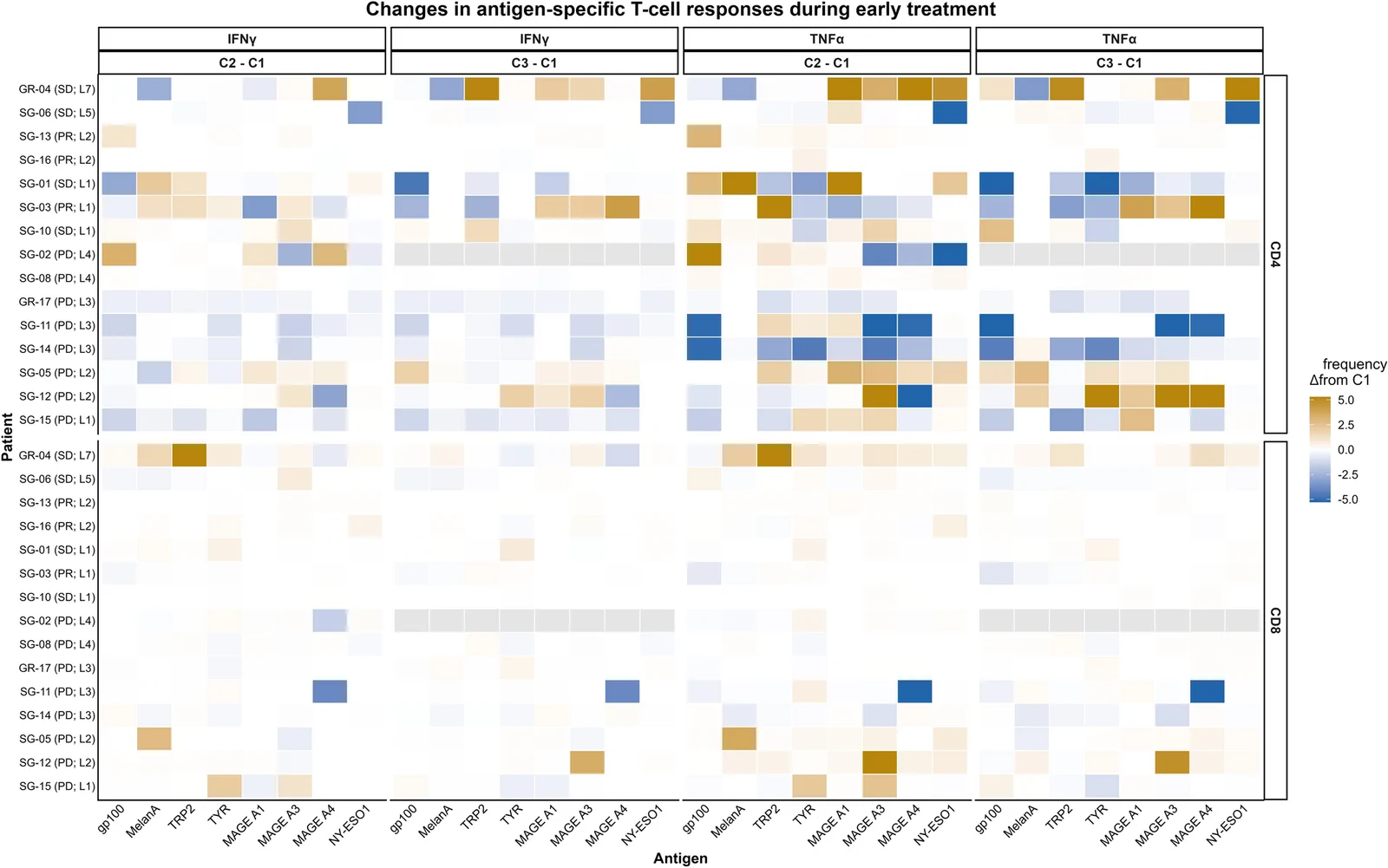
Changes in antigen-specific T cell responses during tinostamustine–nivolumab treatment

Peripheral T cells from multiple patients demonstrated detectable antigen-specific responses following treatment, indicated by increased IFNγ and TNFα production upon stimulation with both melanocyte differentiation antigen (MDAs) and cancer-testis (CT) antigens. Induction of antigen-specific responses was observed at both early treatment timepoints (C2 and C3), irrespective of eventual clinical response status or treatment line of the patients. Overall, CD4+ T cell responses appeared more prominent than CD8+ responses, with TNFα production representing the dominant cytokine signature across patients and antigen classes. Decreases in cytokine-producing T cells were also observed in several patients, in both non-progressors and progressors (Fig. 4). Longitudinal dynamics of antigen-specific T cell responses was also shown for PR, SD, PD (Supplementary Fig. S5) as well as for treatment naïve versus pre-treated groups (Supplementary Fig. S8).

Heatmaps display background-corrected frequencies of cytokine-producing T cells changes relative to baseline (C1), shown as C2-C1 and C3-C1 differences. Increases are shown in gold and decreases in blue on a diverging colour scale centred at zero and capped at ± 5 percentage points; grey cells indicate missing samples. Rows represent individual patients, labelled with best response (PR, SD or PD) and the treatment line in which study treatment was given (L1, first line, i.e. treatment-naïve). Columns represent antigen-specific stimulations including melanocyte differentiation antigens (gp100, MelanA, TRP2, TYR) and cancer-testis antigens (MAGE-A1, MAGE-A3, MAGE-A4, NY-ESO1). Panels are stratified by cytokine production (IFNγ or TNFα) and T cell subset (CD4+ or CD8+).

### Tinostamustine–nivolumab treatment is associated with antigen-specific humoral immune responses

**Fig. 5:**
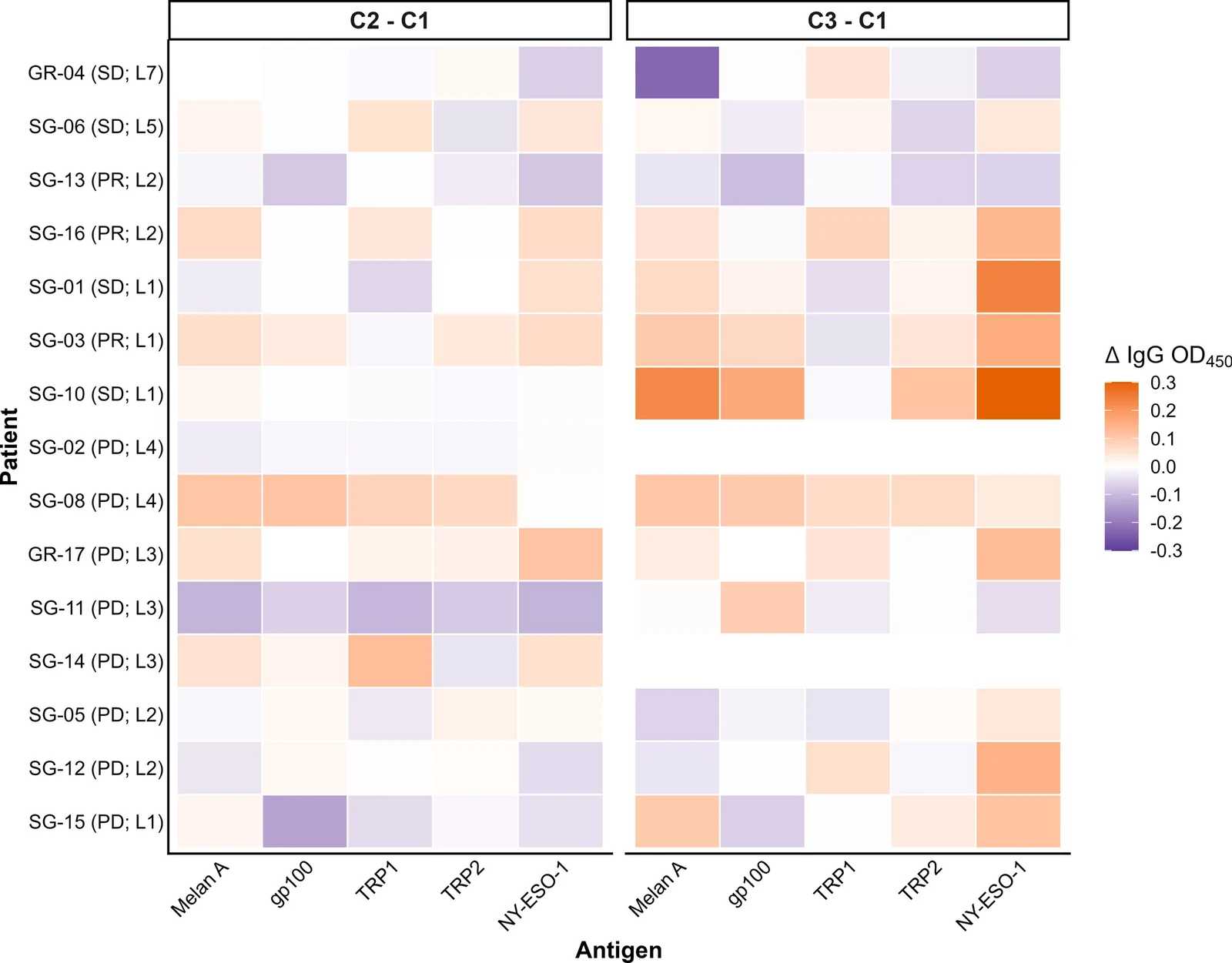
Changes in antigen-specific IgG responses during tinostamustine–nivolumab treatment

Antigen-specific IgG levels evaluated at C2 and C3 relative to baseline (C1) showed that several patients demonstrated increased humoral responses following treatment. Increased delta IgG values were observed in non-progressors against multiple melanoma-associated antigens, including MelanA, gp100, TRP1, TRP2, and NY-ESO1, and were most pronounced at C3. Enhanced humoral responses were detectable at both C2 and C3 timepoints, suggesting ongoing induction or expansion of antigen-specific B cell responses during therapy (Fig. 5). We observed no marked differences in antigen-specific IgG between the response groups PR, SD and PD (Supplementary Fig. S4) as well as treatment-naïve and pre-treated patients at C1– C10 (Supplementary Fig. S7).

Heatmap representation of longitudinal changes in serum melanoma antigen-specific IgG responses following tinostamustine–nivolumab therapy. Heatmaps display changes in ELISA absorbance values (OD450) relative to baseline (C1), shown as C2-C1 and C3-C1 differences. Increases are shown in orange and decreases in purple on a diverging colour scale centred at zero; blank cells indicate missing samples. Rows represent individual patients, labelled with best response (PR, SD or PD) and the treatment line in which study treatment was given (L1, first line, i.e. treatment-naïve). Columns represent melanoma-associated antigens including MelanA, gp100, TRP1, TRP2, and NY-ESO1.

Swimmer plot of treatment duration in weeks for each of the 15 patients in the translational cohort. Bars are coloured by best response according to RECIST v1.1: partial response (PR, red), stable disease (SD, green) or progressive disease (PD, blue). Patients are ordered by response group and then by treatment duration. The adjacent panel shows, for each patient, the number of prior lines of palliative systemic treatment (Prior lines), whether anti-PD-1 therapy was received before enrolment (Prior anti-PD-1) and the timing of that exposure (PD-1 timing): naïve (no prior anti-PD-1), immediate (anti-PD-1 was the line directly preceding study treatment) or remote (anti-PD-1 was received earlier, with at least one intervening line) progressor.

**Fig. 6:**
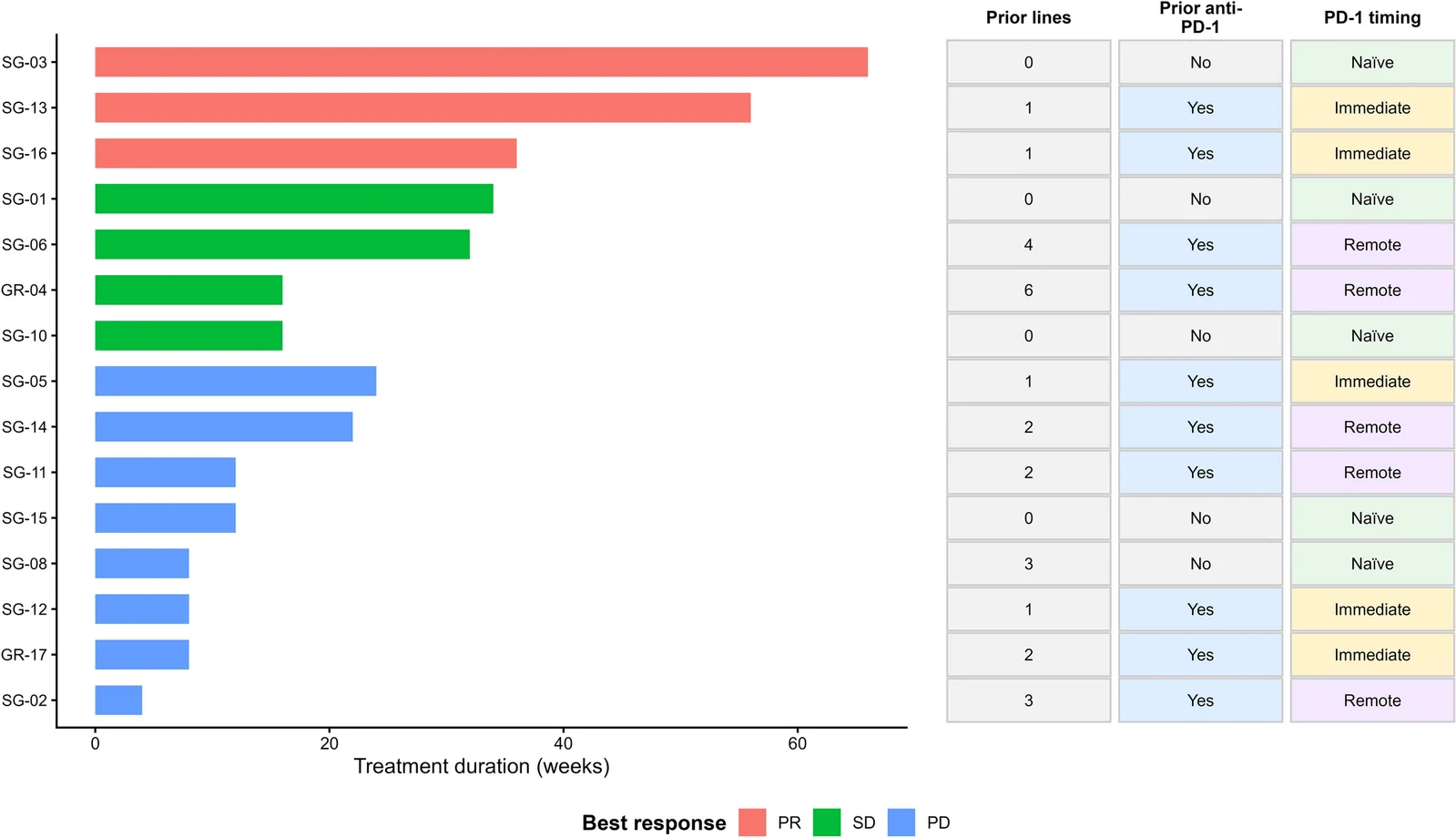
Summary of treatment duration and prior treatment history of patients during tinostamustine–nivolumab therapy

To place the immune findings in clinical context, Fig. 6 summarizes treatment duration, best response, the number of prior treatment lines and the anti-PD-1 history of each patient. Treatment duration ranged from 4 to 66 weeks; the three patients with PR remained on treatment longest (36 to 66 weeks), followed by patients with SD (16 to 34 weeks) and PD (4 to 24 weeks). Antigen-specific T cell and IgG increases (Figs. 4 and 5) were detected in patients with PR, SD and PD and across the range of prior treatment histories, including a patient with six prior treatment lines and patients whose immediate prior therapy was anti-PD-1.

## Discussion

Prior lines of chemotherapy, targeted therapy, and continued disease progression are frequently associated with progressive immune dysfunction and depletion of functional lymphocyte reserves, thereby limiting the capacity of patients to mount effective anti-tumour immune responses [13]. In advanced-stage cancers, chronic antigen exposure and sustained systemic inflammation have additionally been linked to T cell exhaustion and irreversible immune dysfunction [14]. Consequently, heavily pre-treated patients are often considered immunologically compromised, raising important questions regarding their ability to generate meaningful adaptive immune responses during subsequent therapies. In this context, longitudinal translational immune analyses performed during treatment may provide valuable insights into the functional immune competence and systemic immune dynamics of individual patients undergoing therapy.

To our knowledge, this is the first data to show immunomodulatory doses of tinostamustine and nivolumab to be associated with antigen-specific adaptive immune activation in heavily pre-treated, advanced melanoma patients. One of the most notable findings of this study was the induction of antigen-specific T cell responses following treatment. Longitudinal ex vivo stimulation assays demonstrated detectable increases in cytokine-producing T cells directed against both MDAs and CT-antigens. Interestingly, CD4+ T cell responses appeared more prominent than CD8+ responses, with TNFα production representing the dominant cytokine signature across patients. Several patients also demonstrated treatment-associated increases in melanoma-associated antigen-specific IgG responses. These increases were observed in patients across a range of prior treatment histories, including a patient with six prior treatment lines, which supports the retention of adaptive immune competence despite extensive prior therapy. Additionally, longitudinal peripheral immune profiling revealed relatively stable overall leukocyte and neutrophil counts throughout treatment, with descriptive group-specific differences in lymphocyte and monocyte counts, NLR and LMR, whereas progressors demonstrated persistently elevated LDH and S100 levels, consistent with progressive disease and increased systemic inflammatory burden. Notably, elevated baseline LDH in progressors correlated with reduced circulating T cell frequencies and relative enrichment of monocyte populations, suggesting that systemic immune composition may reflect tumour-driven immune dysregulation.

Several limitations of this study should be acknowledged. The exploratory nature of this translational analysis and the small cohort size limited statistical power for certain longitudinal comparisons. Because patient numbers decreased at later treatment cycles due to treatment discontinuation and disease progression, later-cycle longitudinal trends should be interpreted descriptively and within the exploratory nature of this translational analysis. Additionally, peripheral blood analyses may not fully capture immune dynamics within the tumour microenvironment. The translational cohort was heterogeneous with respect to prior therapy: four of 15 patients were treatment-naïve and five were anti-PD-1 naïve, and the exploratory comparison with previously treated patients was too small to support formal statistical conclusions. We therefore describe the cohort as predominantly, rather than uniformly, pre-treated. Prior anti-PD-1 exposure was also heterogeneous (immediate prior line in five patients, remote exposure in five), which probably reflects different states of immune refractoriness, but the cohort was too small to compare these subgroups. All four patients with stable disease had a progression-free duration of more than three months (15.4–34.1 weeks), but only one exceeded six months (Supplementary Table S1). Finally, the antigen-specific analyses focused on C1 to C3, as sample availability was greatest at these timepoints and the later timepoints were available for only a few patients and were not suitable for formal analysis. Nevertheless, the longitudinal design, integration of functional T cell assays, humoral immune profiling, and detailed immune phenotyping provide a comprehensive overview of systemic immune responses during tinostamustine–nivolumab therapy.

Overall, this study demonstrates that majority of pre-treated advanced melanoma patients retain the capacity to mount detectable adaptive immune responses during treatment with immunomodulatory dose tinostamustine and nivolumab.

## Supplementary Information

Below is the link to the electronic supplementary material.

Supplementary file1 (XLSX 15 KB)

Supplementary file2 (PNG 49550 KB)

Supplementary file3 (PNG 320 KB)

Supplementary file4 (PNG 2324 KB)

Supplementary file5 (PNG 2013 KB)

Supplementary file6 (PNG 4233 KB)

Supplementary file7 (PNG 2072 KB)

Supplementary file8 (PNG 1839 KB)

Supplementary file9 (PNG 3087 KB)

## Data Availability

No datasets were generated or analysed during the current study.
